# The role of interleukin‐22 in mammalian intestinal homeostasis: Friend and foe

**DOI:** 10.1002/iid3.1144

**Published:** 2024-02-16

**Authors:** Hedi‐Britt Klotskova, Evelien Kidess, Adria L. Nadal, Sylvia Brugman

**Affiliations:** ^1^ Host Microbe Interactomics, Animal Sciences Group Wageningen University and Research Wageningen The Netherlands

**Keywords:** cytokines, inflammation, molecules, molecular biology, mucosa, processes, techniques/approaches, tissues

## Abstract

Interleukin‐22 (IL‐22) is an important cytokine in the intestinal environment. IL‐22 is mainly produced by immune cells and targeted at nonimmune cells such as epithelial and stromal cells in a broad array of tissues such as ‐but not restricted to‐ the liver and adipose tissue. IL‐22 therefore connects immune functions with metabolic functions of the host, and since it is induced by the microbiota, connects host functioning to the outside environment. IL‐22 induces epithelial cell proliferation aiding in rapid epithelium regeneration and wound healing. Additionally, IL‐22 activates antiapoptotic genes and DNA damage response pathways, enhancing epithelial cell survival. Recently, it has also been shown that IL‐22 induces Paneth cell differentiation in humans. However, IL‐22 can also contribute to intestinal epithelium damage and reduces microbial diversity in the intestine directly or indirectly by inducing excessive antimicrobial peptide production by epithelial cells. Moreover, IL‐22 enhances angiogenesis and may therefore support tumorigenesis in the intestine. In conclusion, it appears that whether IL‐22 has a beneficial or harmful effect in the mammalian intestine largely depends on its regulation. This review aims to provide a comprehensive overview of the current literature and emphasizes that IL‐22 signaling outcome depends on the timing and duration of IL‐22 production, the presence of it regulators such as IL‐22BP, and the specific location of the cytokine production in the gastrointestinal tract.

## Graphical Abstract

Beneficial and harmful effects of IL22 in the intestine. IL‐22 is produced by various immune cells at the intestinal epithelium. Here, it acts on enterocytes by binding to the IL‐22 receptor complex to initiate its effects. These include inducing cell proliferation/tumorigenesis, wound healing, and induction of mucins by Goblet cells and antimicrobial peptides by Paneth cells. If produced in excessive amounts, these antimicrobial peptides may damage the intestinal epithelium and reduce microbial diversity. IL‐22 induces tight junction proteins, modulating epithelial barrier permeability. IL‐22 also induces Paneth cells differentiation in humans (image created with Biorender).

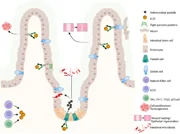

## 1 INTRODUCTION

The human intestine has a large surface area of ~32 m^2^,^1^ whereas the murine intestinal tract has been assessed to be close to 2 m^2^.^2^ The intestine is home to trillions of microorganisms, collectively termed microbiota. The microbiota is separated from the hosts' immune system by a single layer of intestinal epithelial cells. There is a sophisticated communication between the microbiota and the immune system through the epithelial barrier, and this communication is a key for maintaining intestinal homeostasis. Disruption in this communication in the intestine may lead to dysbiosis. Dysbiosis is defined as a disturbance in microbial community due to environmental factors such as food, antibiotic, and medicine, or internal host factors such as overreacting or insufficient immune responses. Intestinal epithelial cells are much more than just a physical barrier between the host and the outside world. These cells secrete mucus and antimicrobial peptides (AMPs), sense harmful and beneficial microbes, and induce and modulate immune responses. This is accomplished by various cell types found in the intestinal epithelium, such as, but not limited to goblet cells and Paneth cells.^3^ Intestinal epithelial cells are connected by tight junction proteins, that maintain the intestinal barrier and regulate paracellular permeability. Another important function of the intestinal epithelium is to provide the host with nutrients.^4^ To perform their cell‐specific functions, intestinal epithelial cells require distinct signals. These signals are often communicated by cytokines and one of these cytokines is interleukin‐22 (IL‐22).

IL‐22 belongs to a family of IL‐10 cytokines. IL‐22 is produced by various immune cells, from both innate and adaptive immunity. Known sources of IL‐22 are T‐helper 1 cells, T‐helper 17 cells, T‐helper 22 cells, γδ T cells, natural killer T cells, and type 3 innate lymphoid cells (ILC3s).^5^ IL‐22 production and bioactivity can be regulated positively as well as negatively (Box 1). Important described positive regulators of IL‐22 production include IL‐23, IL‐1β, IL‐7, aryl hydrocarbon receptor (AhR), and Notch.^5^ Known inhibitors of IL‐22 production and activity include IL‐22 binding protein (IL‐22BP), transforming growth factor‐β, ICOS, c‐Maf, IL‐27, and IL‐25.^5^ The active secreted form of human IL‐22 consists of a 146 amino acid protein that binds to membrane‐bound IL‐22 receptor (IL‐22R), a heterodimeric receptor with IL‐22R1 and IL‐10R2 subunits.^5^ The IL‐10R2 subunit is shared with other cytokines, such as IL‐10, IL‐26, IL‐28, and IL‐29.^5^ IL‐22R1 is found to be expressed on epithelial cells in a number of human tissues, such as skin, small intestine, colon, kidney, as well as pancreas.^6^ However, IL‐22R1 is not found to be expressed on immune cells.^6^ The fact that IL‐22 is produced by immune cells but it exclusively communicates to various nonimmune tissues makes IL‐22 signaling particularly interesting. IL‐22 has been observed to be beneficial as well as harmful in the intestine, indicating that the lack or the presence of IL‐22 may define the gut homeostasis.

Box 1. **Conditions that may determine the outcome of IL‐22 signaling in the intestine**

**Table:** 

Presence and expression of positive regulators (AhR, IL‐23, IL‐1B, IL‐7, Notch, butyrate). Presence and expression of negative regulators (IL‐22BP, TGF‐B, ICOS, c‐Maf, IL‐25 and IL‐27, IL‐10). IL‐22R1/IL10R2 expression. Cell source of IL‐22 (innate vs. adaptive immune cells). Localization of IL‐22 producing cells and range of action of the IL‐22 (local in small intestine or colon; or systemic). Timing, duration and amount of IL‐22 production. Diseased versus homeostatic state (see Table 1).

IL‐22 has been reported to stimulate epithelial cell proliferation and production of mucins—the major components of mucus.^7^, ^8^ Additionally, IL‐22 has been shown to induce Paneth cell differentiation as well as AMP secretion by Paneth cells.^7^ Contrastingly, IL‐22 has also been linked to changes in the tight junction permeability and is therefore thought to disrupt the intestinal barrier and homeostasis.^8^, ^9^ These opposing findings illustrate that the role of IL‐22 in intestinal homeostasis is not yet clearly defined, and it is a matter of ongoing research. This review aims to elaborate on the beneficial as well as harmful effects of IL‐22 in the mammalian intestine, as well as investigate the factors that define its effects on the intestinal homeostasis such as timing, concentrations, location, and regulators.

## 2 BENEFICIAL ROLE OF IL‐22 IN INTESTINAL HOMEOSTASIS

The integrity of the intestinal barrier is essential for a healthy gut. There are examples of intestinal diseases in which the criteria for homeostatic barrier functioning are not met, such as inflammatory bowel disease (IBD), such as ulcerative colitis (UC) and Crohn's disease (CD), or necrotizing enterocolitis (NEC). These multifactorial conditions are usually characterized by excessive or insufficient immune responses leading to unresolved inflammation and disruption in the intestinal epithelial barrier,^10^ alongside other pathological symptoms.^11^, ^12^ The multifactorial nature makes these pathologies difficult to treat and there is, therefore, active ongoing research to find possible therapeutic agents, that could help restore the epithelial barrier in the affected individuals. Enhancing IL‐22 signaling has been suggested as such a therapeutic agent since IL‐22 appears to be a communication bridge between the immune and tissue‐resident nonimmune cells, such as intestinal epithelial cells.^6^ Here, the examples of how IL‐22 may positively influence intestinal homeostasis will be elaborated on.

### 2.1 IL‐22 and mucus

Another way IL‐22 may be beneficial is by inducing mucus secretion. The mucus in the intestine provides a vital protective layer separating gut microbiota from the epithelial cells and the host's immune system, and it helps maintain the integrity of the intestinal barrier.^13^ Commensal and pathogenic bacteria get trapped in the mucus and are expelled from the intestine by peristalsis, preventing excessive bacterial colonization.^14^ Evidence suggests that IL‐22 might play a role in maintaining the mucus layer. In both murine in vivo and human in vitro experiments treatment with IL‐22 increased the expression of membrane‐bound mucins.^8^ Membrane‐bound mucins are expressed on the apical side of intestinal epithelial cells, where they form a glycocalyx (a layer consisting of glycoproteins and glycolipids) by extending 200–1500 nm above the cell surface.^14^ Murine in vivo experiments showed that intravenous injections with IL‐22‐Fc lead to an increase in Mucin‐1 in the colon.^8^ IL‐22‐Fc in this experiment is a murine recombinant IL‐22 linked to the Fc (fragment crystallizable) region of mouse IgG1. Fc‐fusion proteins are used to prolong the plasma half‐life of proteins and therefore increase their therapeutic efficacy.^15^ In vitro experiments with human colon‐derived organoids showed that treatment with recombinant human IL‐22 increases the gene expression of membrane bound mucins MUC1, MUC4, and MUC13.^8^ IL‐22 treatment has also shown to increase MUC1  expression in human jejunum‐ and duodenum‐derived organoids.^7^ However, in mice, the expression of *Mucin‐2* and the presence of goblet cells was not altered upon treatment with IL‐22 in the colon.^8^ It is possible that the effect of IL‐22 on the expression of mucins may differ between the different sections of intestine. Human and mouse membrane‐bound mucins have been shown to stimulate cell migration and inhibit apoptosis.^14^ The evidence that IL‐22 upregulates the expression of some of the membrane‐bound mucins in human epithelial cells suggests that treatment with IL‐22 may have a beneficial effect in conditions where patients are challenged with increased bacterial burden or a disrupted epithelial barrier, such as IBD or NEC.

### 2.2 IL‐22 and intestinal barrier function

The intestinal epithelial barrier is protected by AMP production as well as rapid epithelial cell renewal. The gastrointestinal epithelium is renewed in 2–3 days in mice and in 3–5 days in humans.^4^ The rapid regenerative capacity of the intestinal epithelium allows for a symbiotic relationship between the host and the microbiota within the gut. Intestinal stem cells (ISCs) are the sole source of all epithelial cell types found in the intestine.^16^ It is therefore essential that the ISCs are well protected from the possibly harmful luminal contents, and that these cells receive correct signals to appropriately differentiate and proliferate.

ISCs are offered protection by their location deep within the crypts, the AMPs produced by neighboring Paneth cells, and the mucus layer.^16^ IL‐22 appears to initiate signals for ISC and epithelial cell proliferation, differentiation, and functioning. A number of in vivo and in vitro studies have shown that IL‐22 might have the capability to induce the proliferation of epithelial cells, but also stem cells in the intestine.^8^, ^17^, ^18^, ^19^ In case of colonic epithelial cells, it has been shown that treatment with recombinant human IL‐22 leads to epithelial cell proliferation in human primary colon tissue organoids.^8^ In agreement with this, Patnaude et al.^8^ also reported that by injecting mice intravenously with IL‐22‐Fc, colon epithelial cells exhibit increased proliferation. IL‐22 positively affects the stem cell compartment in the intestine. For example, treating murine small intestine‐derived organoids with exogenous recombinant IL‐22 increases organoid size by inducing Lgr5+ stem cell proliferation.^17^ Likewise, administering mice bacteria carrying IL‐22 increased Lgr5+ stem cells in the ileum.^19^ However, others have not been able to replicate these effects on lgr5+ stem cells.^7^, ^11^, ^18^, ^20^ Instead, Zha et al.^18^ observed increased proliferation of transit‐amplifying (TA) cells upon IL‐22 treatment leads of jejunal enteroids. TA cells are a cell type in a developmental stage between stem cells and fully differentiated epithelial cells.^21^ Likewise, by using IL‐22 transgenic mice it was shown that IL22 increased the proliferative capacity in the TA cell compartment.^20^ Zwarycz et al.^20^ suggested that IL22 might shift the balance of symmetric self‐renewal to asymmetric division where ISC division produces one ISC and one TA progenitor. However, He and colleagues, failed to see epithelial proliferation (of neither the ISC or TA compartment) in their human organoid culture.^7^ The effect of IL‐22 on epithelial proliferation, therefore, remains unclear.

Recently, IL‐22 was shown to drives the differentiation of Paneth cells, the major producers of AMPs, in human small intestine organoids.^7^ Adding to the existing body of evidence that IL‐22 induces AMP production in the intestine.^7^, ^8^, ^11^, ^17^ In vitro experiments with small intestine tissue from humans and mice have shown that treatment with IL‐22 increases the messenger RNA (mRNA) levels of different AMPs, such as *REG3β* and *REG3γ*.^11^, ^17^ Additionally, *REG1α, REG1β, REG3α, β‐DEFENSIN 3*, and *LIPOCALIN‐2* gene expression has been observed to be upregulated in human colonic organoids, as well as small intestine‐derived organoids when treated with IL‐22.^7^, ^8^ The aforementioned in vitro studies are also supported by results from murine in vivo experiments, where *Reg3β* and *Reg3γ* expression was upregulated in the colon after treatment with IL‐22.^8^, ^17^, ^22^ The abovementioned studies illustrate that IL‐22 can be beneficial in maintaining the intestinal homeostasis. The presence of IL‐22 in the intestine can lead to AMP production and thus protect stem cells. Furthermore, it might enhance cell renewal in the small and large mammalian intestine to allow for rapid epithelial cell renewal.

### 2.3 IL‐22 and colitis

One disease where IL‐22 may have a positive influence is NEC. NEC is an intestinal inflammatory disorder primarily affecting neonates and premature infants, and it is thought to occur because of an underdeveloped immune system.^23^ A mouse study found fewer IL‐22‐producing cells as well as low levels of IL‐22 in the intestinal lamina propria of neonate pups compared with adult mice.^11^ Mihi et al.^11^ also showed that daily intraperitoneal injections with recombinant murine IL‐22 for 3 days led to a reduction in pro‐inflammatory cytokine *Il1*β as well as in reduction in disease severity in NEC‐induced pups compared with phosphate‐buffered saline‐treated littermates. Treatment with IL‐22 was further shown to decrease the damage of ileal epithelium in mice.^11^ Reduction in pro‐inflammatory cytokine profile and intestinal damage during in vivo experiments indicates that IL‐22 treatment may have restorative effects in the treatment of NEC by reducing excessive inflammation and enhancing epithelium healing.

IL‐22 has been shown to be protective in a colitis model applied in mice. This colitis model uses a transfer of CD4^+^CD45RB^hi^ T cells (naïve T cells) into *Rag1* ^*−/−*^ mice to induce intestinal disease. As these *Rag1^−/−^* mice lack all T cells (including regulatory T cells), there is no regulation of the transferred T cells and these cells rapidly increase in numbers, causing inflammation in the intestine. This *Rag1^−/−^* model has been employed to study the role of IL22 in colitis. For example, *Rag1* ^*−/−*^ or *Il22* ^*−/−*^ *Rag1* ^*−/−*^ mice received IL‐22‐deficient or wild‐type (WT) CD4^+^CD45RB^hi^ T cells intraperitoneally, and although all mice developed colitis, the mice fully lacking IL‐22 developed most severe symptoms.^24^ This suggests that IL‐22 of both innate and adaptive origin had a protective role in this murine colitis model. Recently, it has also been shown that modulation of tryptophan metabolism via the AHR‐IL22 pathway could mediate the alleviation of dextran sulfate sodium (DSS)‐induced colitis.^25^ Furthermore, IL‐22 ameliorated DSS‐induced UC via activation of autophagy and subsequent induction of occluding expression.^26^

### 2.4 IL‐22 and short‐chain fatty acids

Short‐chain fatty acids are products of anaerobic fiber fermentation by the microbes in the colon and are the main source of energy for intestinal colonocytes.^27^ In vitro studies using Caco2 cells have shown that butyrate can influence the IL‐22 pathway by upregulating *IL‐22R1* and directly amplify signal transducer and activator protein (STAT)3‐mediated gene activation.^28^ Similarly, addition of butyrate to human colon‐derived organoids increased MUC13 expression and its effects were further enhanced when IL‐22 was added together with butyrate.^8^ However, the increase of MUC1 expression induced by IL‐22 was not further enhanced by coadministration of butyrate, indicating that butyrate and IL‐22 have both unique and synergistic effects on the expression of membrane‐bound mucins and AMPs (recently, research performed in zebrafish uncovered a link between acetate produced by microbes and upregulation of IL22).^29^ Specifically, this study showed that vitamin D induced AMP expression by activating IL‐22 signaling by increasing the abundance of acetate‐producing bacteria.^29^ The mechanism by which acetate might increase IL‐22 signaling, however, remains to be investigated.

### 2.5 IL‐22 as therapeutic agent

As mentioned above, IL‐22 can lead to proliferation of intestinal epithelial cells and could thus aid in the regeneration of the intestinal epithelium upon damage. The effects of IL‐22 as a therapeutic agent have been investigated in the context of irradiation damage in the gut. Irradiation causes damage to different intestinal cell types, such as γδ T‐cells and helper T‐cells, but also ISCs, Paneth cells, and goblet cells.^19^ γδ T‐cells and helper T‐cells are known sources of IL‐22.^5^ Zhang et al.^19^ presented a novel approach to deliver IL‐22 to the site of intestinal damage. They transformed *Lactobacillus reuteri* and *Escherichia coli* to carry recombinant plasmids with murine IL‐22, which would be expressed by the bacteria in the intestine. They showed that treatment with *L. reuteri* and *E. coli* carrying the IL‐22 transgene can increase the survival of mice up to 85%, compared with the control group, if administered by gavage 24 h after total body irradiation. An important advantage of using *L. reuteri* as a vehicle to deliver the biotherapeutic agents to the intestine is because this bacteria is considered safe for the host and can be transformed to additionally carry prophages that would lyse the bacteria during the gastrointestinal transit, after which the therapeutic agent *L. reuteri* is carrying would be released.^30^ It is important to bear in mind that IL‐22 delivery to the intestine by bacteria carrying the IL‐22 transgene would likely be beneficial only in cases when the epithelial barrier is severely disrupted, as the receptors for IL‐22 are found on the basolateral side of the epithelial cells and not on the apical side,^8^, ^9^ and IL‐22 could thus not elicit its effects from the gut lumen.

Furthermore, IL22 could be used to combat genotoxic stress. The intestine is challenged daily by potentially pathogenic bacteria and possibly genotoxic food items ingested by the host. Genotoxicity is the ability of a substance to damage DNA, and possibly lead to mutations and tumors. One such source of genotoxic stress is glucosinolate‐containing cruciferous vegetables, such as broccoli and cabbage.^31^ Although glucosinolates themselves are not genotoxic to mammalian cells, some of their breakdown products (by bacterial enzymes), such as 1‐methoxy‐3‐indolylmethyl alcohol (1‐MIM‐OH), can be.^31^ A study demonstrated that stimulating murine intestinal cells in vivo by gavage with 1‐MIM‐OH led to an increase in DNA adduct formation in the cecal tissue compared with the controls.^22^ DNA adducts are formed when substances bind to DNA, leading to nucleotide mispairings and eventually mutations. Remarkably, it was shown that 1‐MIM‐OH treatment increased IL‐22^+^ ILC3s and γδ T cells, as well as IL‐22 production by these cell types.^22^ 1‐MIM‐OH treatment also led to an increase in the expression of a known IL‐22 response gene, *Reg3γ*, in primary colon epithelial cells in vivo.^22^ This experiment shows that even though glucosinolate metabolites may have a genotoxic effect on intestinal cells, they significantly increase the amount of IL‐22^+^ cells as well as IL‐22 in the intestine. It is noteworthy that the concentrations of 1‐MIM‐OH used in the experiment are considerably higher than the average estimated daily intake of glucosinolates in men and women.^31^ Thus, humans would have to consume kilograms of cruciferous vegetables per day to attain harmful concentrations of glucosinolate metabolites in their intestines. Nevertheless, IL‐22 may protect against genotoxic stress by inducing DNA damage response (DDR) pathways.^22^ DDR is a safety mechanism containing a network of activated genes that protect the cells from accumulating mutations when challenged with genotoxic stress. Mice subjected to a glucosinolate‐free diet exhibited a significant reduction in DDR gene expression, as well as IL‐22 production in their primary colon epithelial cells.^22^ On the contrary, DDR effector gene expression and IL‐22 production were upregulated in mice who received glucosinolate‐containing diet.^22^ These results indicate that IL‐22 may be necessary for an appropriate initiation of DDR in the mammalian intestine.

Taken together, IL‐22 appears to participate in many processes involved in maintaining intestinal homeostasis. It has been shown to protect against genotoxic stress, decrease the damage to ileal epithelium, and decrease the expression of pro‐inflammatory cytokine during induced NEC. IL‐22 has also been demonstrated to increase the production of membrane‐bound mucins as well as AMPs by intestinal epithelial cells. Additionally, it has been reported that IL‐22 can increase the proliferative capacity of intestinal stem and epithelial cells. All the above‐mentioned processes are necessary for the maintenance and restoration of intestinal homeostasis, and it appears that IL‐22 can shift the homeostasis in the mammalian gut towards a positive direction (Table 1). However, IL‐22 has also been demonstrated to have detrimental effects in the intestine.

**Table 1:** Beneficial effects of IL‐22 signaling.

Effect	Location	State	Model	Concentration	In vivo/in vitro	Reference
Reduction of IL‐1β (pro‐inflammatory cytokine) during NEC	Ileum	Diseased	C57BL/6 mice	100 µg/kg	In vivo	Mihi et al.^11^
Protection during induced colitis	Colon	Disease‐prone	*Il22* ^*−/−*^ *Rag* ^*−/−*^ mice, *Rag* ^*−/−*^ mice	Transfer of *Il22* ^*+/+*^ or *Il22* ^*−/−*^ CD4^+^CD45RB^hi^CD25^−^NK1.1^−^ cells (5 × 10^5^ cells)	In vivo	Zenewicz et al.^24^
Decrease in epithelial damage and disease severity	Ileum	Diseased	C57BL/6 mice	100 µg/kg	In vivo	Mihi et al.^11^
Increased mucins	Colon	Healthy	C57BL/6N mice	2.5 mg/kg	In vivo	Patnaude et al.^8^
	Colon	Healthy, diseased	Human colon‐derived organoids	10 nM	In vitro	Patnaude et al.^8^
	Ileum, duodenum	Healthy	Human ileum‐ and duodenum‐derived organoids	2 ng/mL	In vitro	He et al.^7^
Increased epithelial cell proliferation	Colon	Healthy	Human colon‐derived organoids	1.2 nM	In vitro	Patnaude et al.^8^
	Colon	Healthy	C57BL/6 N mice	2.5 mg/kg	In vivo	Patnaude et al.^8^
	Small intestine	Healthy	Murine small intestine‐derived organoids	1 ng/mL	In vitro	Lindemans et al.^17^
	Jejunum, ileum	Healthy	C57BL/6 mice	1 µg/mL	In vivo	Zha et al.^18^
	Jejunum	Healthy	Murine jejunal enteroids	5 ng/mL	In vitro	Zha et al.^18^
Increased stem cell proliferation	Small intestine	Healthy	Murine small intestine organoids	1/5 ng/mL	In vitro	Lindemans et al.^17^
	Colon	Healthy	Human colon‐derived organoids	1.2 nM	In vitro	Patnaude et al.^8^
	Ileum	Diseased	C57BL/6 mice	100 μL saline solution containing *L. reuteri*‐IL‐22 or *E. coli*‐IL‐22	In vivo	Zhang et al.^19^
Increased TA‐cell proliferation	Jejunum	Healthy	Murine jejunal enteroids	5 ng/mL	In vitro	Zha et al.^18^
Paneth cell differentiation	Ileum, duodenum	Healthy	Human ileum‐ and duodenum‐derived organoids	2 ng/mL	In vitro	He et al.^7^
Increased AMP production	Ileum, duodenum	Healthy	Human ileum‐ and duodenum‐derived organoids	2 ng/mL	In vitro	He et al.^7^
	Small intestine	Diseased	C57BL/6 mice	4 µg/day	In vivo	Lindemans et al.^17^
	Small intestine	Healthy	Murine small intestine enteroids	100 ng/mL	In vitro	Mihi et al.^11^
	Ileum	Healthy	Human ileum‐derived enteroids	100 ng/mL	In vitro	Mihi et al.^11^
	Ileum	Healthy, diseased	C57BL/6 mice	100 µg/kg	In vivo	Mihi et al.^11^
	Colon	Healthy, diseased	Human colon‐derived organoids	0.001–20 nM	In vitro	Patnaude et al.^8^
	Colon	Healthy	C57BL/6N mice	5 mg/kg	In vivo	Patnaude et al.^8^
	Colon	Diseased	*Il22* ^+/+^ and *Il22* ^−/−^ mice	‐ (*Il22* ^*+/+*^ vs *Il22* ^*−/−*^)	In vivo	Gronke et al.^22^
Improved survival of mice after total body irradiation	Entire organism	Diseased	C57BL/6NTac mice	100 μL saline solution containing *L. reuteri*‐IL‐22, or *E. coli*‐IL‐22	In vivo	Zhang et al.^19^
Enhanced DDR	Colon	Diseased	*Il22* ^+/+^ and *Il22* ^−/−^ mice	‐ (*Il22* ^*+/+*^ vs *Il22* ^*−/−*^)	In vivo	Gronke et al.^22^

Abbreviations: AMP, antimicrobial peptide; DDR, DNA damage response; IL‐1β, interleukin‐1β; IL‐22, interleukin‐22; NEC, necrotizing enterocolitis.

## 3 HARMFUL ROLE OF IL‐22 IN INTESTINAL HOMEOSTASIS

IL‐22 has several properties that make it suitable as a therapeutic agent due to its role in maintaining intestinal homeostasis. In the next section, however, the focus will be on the harmful effects of IL‐22 in the intestine and why it could be appealing to therapeutically target, in other words inhibit, IL‐22 instead.

### 3.1 IL‐22 and epithelial barrier integrity

Although we have discussed the beneficial effects of IL‐22 in intestinal barrier integrity, some studies indicate that IL‐22 may decrease the barrier integrity. Experiments with epithelial cell monolayers have shown that IL‐22 treatment increases the paracellular permeability and reduces transepithelial electrical resistance (TEER).^8^, ^9^ Reduction in TEER indicates reduced epithelial integrity. Experiments with human colorectal Caco‐2 cells demonstrated that treating the monolayer with recombinant human IL‐22 for 72 h from the basolateral side significantly decreased TEER compared with the control treatment.^9^ To confirm that IL‐22 is responsible for reducing TEER, the monolayer was treated with recombinant human IL‐22BP, a known negative regulator of IL‐22. IL‐22BP treatment counteracted the TEER‐reducing effect of IL‐22 on Caco‐2 cell monolayer.^9^ Similar results were obtained in experiments with the human colon‐derived T‐84 cell line, where a significant decrease in TEER was observed 48 h posttreatment with human recombinant IL‐22.^8^ IL‐22 exhibited no effect on TEER, nor the expression of IL‐22‐inducible genes, such as *REG1A* and *REG3G*, when administered from the apical side of the abovementioned cell lines,^8^, ^9^ illustrating the importance of IL‐22 reaching its receptor on the epithelial cells' basolateral side to elicit its effects. Moreover, cell viability was also not affected by IL‐22 treatment.^8^, ^9^ Interestingly, it was shown that butyrate is able to override the disruptive effects of IL‐22 on TEER.^8^ This indicates once again that the presence of butyrate in the (large) intestine might influence the effects IL‐22 has in the mammalian gut.

IL‐22 is thought reduce epithelial integrity by altering tight junction structures, namely by *CLAUDIN*‐2 upregulation.^8^, ^9^ Claudin‐2 is a junctional protein expressed in the gastrointestinal tract in humans.^9^ Claudin‐2 forms paracellular channels to allow the passage of solutes, such as Na^+^.^32^ However, overexpression of *CLAUDIN*‐2 may lead to excessive transepithelial paracellular leakage in the intestine, that can be harmful to the host. Upregulation of *CLAUDIN*‐2, as well as IL‐22, is seen in intestinal diseases, such as CD and UC.^9^, ^33^ Treating Caco‐2 cells with IL‐22 for 24, 48, 72, and 96 h showed increased expression of *CLDN2*, the gene coding for *CLAUDIN*‐2 protein, but no other measured tight junction protein‐coding genes.^9^ *CLAUDIN*‐2 expression was also upregulated upon IL‐22 treatment in human primary intestinal epithelium cells,^9^ as well as in mice in vivo and human organoids derived from healthy and UC donors.^8^ To demonstrate that *CLAUDIN*‐2 upregulation is indeed induced by IL‐22, co‐treatment of IL‐22 and IL‐22BP completely abrogated the effects of IL‐22 alone on *CLAUDIN*‐2 expression.^9^ Additionally, knocking down the *CLDN2* gene in Caco‐2 cells confirmed that *CLAUDIN*‐2 causes the reduction of TEER.^9^ Taken together, in the experimental setting of epithelial cell monolayers, it has been shown that IL‐22 can be detrimental by upregulating junctional protein *CLAUDIN*‐2 and thereby reducing TEER.

The epithelial integrity in the intestine is essential for proper functioning and the evidence that IL‐22 can harmfully act upon this barrier illustrates that IL‐22 may also be detrimental. However, it is important to realize that these experiments were conducted on cell monolayers and may not reflect physiological conditions present in the mammalian intestine.

### 3.2 IL‐22 and ISCs

Contrastingly to the results presented about the beneficial effect of IL‐22 on ISC proliferation, some studies report IL‐22 reducing the numbers of ISC. Zha et al.^18^ found that treating murine jejunal enteroids with murine recombinant IL‐22 reduces the number of Lgr5+ ISCs. These in vitro results were supported by in vivo experiments. Intraperitoneal injections with IL‐22 for 7 days (1 µg/day) led to a decrease in Lgr5+ stem cell population in jejunum as well as ileum in the treated mice.^18^ Another study reported that IL‐22 treatment reduced human small intestine organoid formation efficiency, organoid budding, and increased cell death.^7^ As ISCs are the source of all types of intestinal epithelial cells,^16^ their maintenance is critical to maintaining intestinal homeostasis. IL‐22 is upregulated in CD and UC,^33^ and the results from the abovementioned studies suggest that too much IL‐22 could be detrimental for intestinal epithelial regeneration and could be one of the factors responsible for impaired mucosal regeneration and impaired healing observed in IBD.^10^

### 3.3 IL‐22 in colitis models

In contrast to the reported beneficial effects of IL‐22 in colitis, some studies using murine colitis models report that IL‐22 has a harmful role in the intestine. In an experiment with *Il‐23* ^*‐/WT*^ *Rag* ^*−/−*^ mice, neutralization of endogenous IL‐22 with anti‐IL‐22 antibody after anti‐CD40 injections leads to a significant reduction in weight loss, colitis scores, and colon pathology.^34^ When IL‐22 levels were restored with IL‐22‐expressing plasmid injections in *Il‐23* ^*−/−*^ *Rag* ^*−/−*^ mice, they again developed severe colitis upon injections with anti‐CD40.^34^ Injection of empty plasmids alone did not lead to colitis development. These observations suggest that in mice, IL‐22 has a pathological effect in anti‐CD40‐induced acute innate colitis. However, colitis developed in *Il‐23* ^*−/−*^ *Rag* ^*−/−*^ mice only after anti‐CD40 injection, indicating that IL‐22 plasmid injections alone do not cause colitis.^34^ Additionally, it was reported that *Il‐23* ^*−/−*^ *Rag* ^*−/−*^ mice who received IL‐22 plasmid after anti‐CD‐40 injections had significantly more neutrophils in the colonic lamina propria compared with empty‐vector recipients. The authors suggested that IL‐22 may facilitate colitis pathology by recruiting neutrophils to the site of intestinal damage.^34^ Neutrophils produce neutrophil extracellular traps (NETs) to bind pathogens, but too many NETs may also be harmful to the host).^35^ Excessive neutrophil recruitment in the intestine may lead to enhanced NET production and induce inflammation in the colon. The experiments by Eken et al.^34^ indicate that IL‐22 on its own is likely not disruptive, but rather it initiates signals that recruit other cell types or cytokines leading to harmful outcomes.

IL‐22 also had a detrimental effect in the T‐cell transfer model of experimental colitis. CD4^+^CD45RB^hi^ T cells (naïve) or T_reg_ cell‐depleted CD4^+^CD45RB^lo^ T cells (memory/effector) derived from WT mice were transferred into *Rag1* ^*−/−*^ mice to induce colitis.^36^ Specifically, Kamanaka et al.^36^ found increased *Il‐17* and *Il‐22* mRNA expression after disease development in the colons of *Rag1* ^*−/−*^ mice who received T_reg_ cell‐depleted CD4^+^CD45RB^lo^ (memory/effector) T cells in comparison to mice who received the naïve CD4^+^CD45RB^hi^ (naïve) T cells. They additionally report that transfer of IL‐22 knockout (KO), but not IL‐17 KO T cells (CD4^+^CD45RB^lo^ T and T_reg_ depleted) to *Rag1* ^*−/−*^ mice reduced weight loss and colitis scores compared with mice who received WT T cells. This indicates that memory/effector T‐cell‐derived IL‐22 might be involved in the pathogenicity of the colitis model used in this study. Possible mechanisms by which IL‐22 can influence colitis is by inducing epithelial hyperplasia as there is evidence that IL‐22 promotes epithelial cell proliferation in the colon.^8^, ^36^ Additionally, the levels of Reg3γ were significantly reduced in IL‐22 KO memory‐effector transfer mice, suggesting that IL‐22 may drive colitis by inducing REG3γ, that may alter the microbiota composition and lead to dysbiosis.^36^ Interestingly, the results of the study by Kamanaka et al.^36^ again emphasize that the source of IL‐22 might be determining the effects in its target tissues in view of the fact that IL‐22 (derived from innate cells) was protective in the CD4^+^CD45RB^hi^ (naïve T‐cell transfer) colitis model.^24^

Another colitis model often used to investigate intestinal inflammation mechanisms is by inhibiting, knocking down, or knocking out IL‐10 in mice. IL‐10 inhibits the expression of pro‐inflammatory cytokines, such as tumor necrosis factor‐α, IL‐6 and interferon‐γ,^37^ and therefore regulates inflammation. Gunasekera et al.^38^ found that IL‐22 levels, as well as a number of antimicrobial IL‐22‐target gene mRNA levels, were significantly higher in *Il‐10^−/−^* mice compared with the WT mice. IL‐22 protein levels were also increased in the colon and small intestine of *Il‐10^−/−^* colitic mice compared with the WT mice.^38^ These observations suggest that IL‐10 negatively regulates IL‐22 expression in the intestine. Since it is known that IL‐22 upregulates AMPs in the intestine, the diversity of microbiota was evaluated in *Il‐10^−/−^* mice. *Il‐10^−/−^* mice had less diverse microbiota compared with WT mice, as well as *Il‐10^−/−^Il‐22^−/−^* and *Il‐22^−/−^* mice.^38^ Reduced microbiota diversity is usually correlated with gut dysbiosis. It is possible that IL‐22 driven AMP upregulation in the intestine leads to dysbiosis and consequently to intestinal disorders. Several Reg‐family AMPs, such as Reg1α/β, Reg3, and Reg4 are known to be overexpressed in the intestines of humans with UC and CD.^39^, ^40^ To determine a more specific role of IL‐22 in this colitis model, *Il‐10^−/−^Il‐22^−/−^* double KO mice were used. *Il‐10^−/−^Il‐22^−/−^* nor *Il‐22^−/−^* mice develop chronic colitis, indicating that IL‐22 is involved in chronic colitis development.^38^ Although *Il‐10^−/−^* colitic mice exhibited rectal prolapse, as well as ulcerations, crypt abscesses, and mucosal hyperplasia in the colon, *Il‐10^−/−^Il‐22^−/−^* and *Il‐22^−/−^* mice did not develop any of the symptoms.^38^ However, it is important to bear in mind that in this study exogenous IL‐22 in *Il‐10^−/−^Il‐22^−/−^* mice was not used to show that indeed IL‐22 is the aberrant factor. Taken together, these data indicate that IL‐10 is an important negative regulator of IL‐22 and its downstream genes in the intestine, and without appropriate regulation, IL‐22 may become aberrant and cause pathology in the intestine.

The detrimental role of IL‐22 is further emphasized by Powell and co‐workers, showing that IL‐22 is involved in the endoplasmic reticulum (ER) stress response.^41^ In active CD patients both IL22‐responsive and ER stress response pathways were enriched in the colon. The authors show that IL22 is an important driver of this colonic ER stress response in the ILC3 and microbiota‐dependent TRUC model of IBD (T‐bet^−/−^, Rag2^−/−^).^41^ Furthermore, in active IBD, the IL‐22 levels in serum are elevated^42^ and in UC patients, enrichment of IL‐22 regulated transcripts in colonic biopsies correlated with neutrophil infiltration in the colon.^43^ Using an organoid system, Pavlidis and colleagues showed that IL‐22‐mediated transcriptional regulation of CXC‐family neutrophil chemokine expression was dependent on STAT3 signaling, and resulted in recruitment of CXCR2+ neutrophils into colonic tissue contributing to pathology.^43^

### 3.4 IL‐22 regulation and its role in tumorigenesis

Although IL‐22 is seen as beneficial in the intestine by promoting epithelial cell proliferation, it may also be detrimental for the same reason. IL‐22 is found excessively expressed in human colon cancer tissues compared to healthy donor tissues, and in vitro experiments have shown that IL‐22 enhances tumor proliferation.^44^ Tumors are formed by uncontrolled cell proliferation, and IL‐22 has been shown to promote epithelial cell proliferation in human and mouse models.^8^, ^17^, ^18^ It is therefore hypothesized that when IL‐22 lacks correct inhibiting signals in the intestine during a steady state, for instance by IL‐22BP, it may initiate tumor formation by signaling epithelial cells to continuously proliferate. IL‐22BP, a potent IL‐22 inhibitor produced by dendritic cells,^45^ is upregulated during homeostatic conditions and is downregulated in the colon upon mechanical damage, whereas IL‐22 levels exhibit the opposite.^46^ It has been shown in murine models that IL‐22 and IL‐22BP exhibit inverse expression patterns.^46^ Additionally, *Il22bp^−/−^* mice showed increased epithelial cell proliferation during the DSS‐induced colitis recovery phase whereas in WT mice, the cell proliferation during the recovery phase had reduced to a rate similar to steady‐state conditions.^46^ Moreover, they showed that lack of IL‐22BP lead to accelerated development and higher number tumors in the colon in comparison with WT mice.^46^ The study by Huber et al.^46^ illustrates how important the tight regulation of IL‐22 is in the intestine. Tumor formation is unquestionably a multifactorial process and IL‐22 alone certainly is not responsible for this process, but without appropriate regulation it may enhance tumorigenesis by initiating excessive epithelial cell proliferation.

IL‐22BP is not only important in preventing IL‐22 signaling in tumor formation but also in continuous regulation of IL‐22 signaling in the intestine of healthy individuals where it helps to maintain homeostasis. IL‐22BP levels are usually upregulated during steady‐state conditions and downregulated during inflammation. It allows to maintain the levels of IL‐22 low in a healthy gut and elevated during intestinal damage. However, both IL‐22 and IL‐22BP have been observed to be increased in human CD and UC.^47^ These observations emphasize that IL‐22 is a potent cytokine that is actively upregulated during inflammation, and that elevated IL‐22BP levels may not always be enough to inhibit IL‐22 signaling.

Although the study by Huber et al.^46^ clearly shows that it is important to appropriately inhibit IL‐22 to suppress tumor formation, they do not elaborate on the mechanisms by which IL‐22 enhances tumor growth. Others have suggested to therapeutically target (and thus inhibit) IL‐22, as there is evidence that it promotes tumor angiogenesis.^48^ Protopsaltis et al.^48^ showed that IL‐22 enhances human endothelial cell proliferation, survival, and migration in a dose‐dependent manner in vitro. Using the ex vivo murine choroid explant model, they also showed that IL‐22 treatment promotes vessel outgrowth significantly more than the controls. Finally, they demonstrated that blocking IL‐22 significantly reduces the volume of tumors induced by EL4 T‐cell lymphoma cell line in *Rag* ^*−/−*^ and C57BL/6 mice in vivo compared to controls. Even though the experiments by Protopsaltis et al.^48^ did not show the harmful effect of IL‐22 in the intestine, their findings could also be relevant to types of cancer found in the intestine since the induction of angiogenesis is one of the hallmarks of cancer.^49^ Human colon cancer patients have been observed to have significantly higher levels of IL‐22 in their cancer tissue than healthy controls.^44^ It is thus possible that IL‐22 enhances intestinal, as well as other tissue, tumor growth and development by promoting angiogenesis.

Taken together, there are many examples of IL‐22 having a negative impact on intestinal homeostasis in mammals, when dysregulated. In vivo and in vitro experiments have demonstrated that IL‐22 leads to a reduction of ISCs and intestinal epithelial barrier integrity, but also enhances paracellular permeability via upregulation of junctional protein claudin‐2. It has also been shown that IL‐22 can exacerbate colitis in some murine models. Lastly, it was shown that IL‐22 promotes tumor angiogenesis by enhancing endothelial cell proliferation, survival, and migration. All studies referring to harmful effects of IL‐22 are summarized in Table 2.

**Table 2:** Harmful effects of IL22 signaling.

Effect	Location	State	Model	Concentration	In vivo/in vitro	Reference
Reduced stem cells	Jejunum	Healthy	Murine jejunum‐derived enteroids	5 ng/mL	In vitro	Zha et al.^18^
	Jejunum, ileum	Healthy	C57BL/6 mice	1 µg/day	In vivo	Zha et al.^18^
Reduced epithelial integrity (TEER)	Cell monolayer	Diseased	T‐84 cells	1 nM	In vitro	Patnaude et al.^8^
	Cell monolayer	Diseased	Caco‐2 cells	100 ng/mL	In vitro	Wang et al.^9^
Upregulation of claudin‐2	Cell monolayer	Diseased	Caco‐2 cells	1‐100 ng/mL	In vitro	Wang et al.^9^
	Small intestine	Healthy	Human primary intestinal epithelium	100 ng/mL	In vitro	Wang et al.^9^
	Colon	Healthy	BALB/c mice	25 µg/animal	In vivo	Wang et al.^9^
	Colon	Healthy, diseased	Human colon‐derived organoids	10 nM	In vitro	Patnaude et al.^8^
Exacerbate colitis (anti‐CD40 injections)	Colon	Diseased	*Il‐23* ^*−/−*^ *Rag* ^*−/−*^ mice	15 µg of IL‐22‐containing plasmid resuspended in 2 ml of saline	In vivo	Eken et al.^34^
Induce colitis	Colon	Disease‐prone	*Rag1* ^*−/−*^ mice	Transfer of T_reg_ cell‐depleted CD4^+^CD45RB^lo^ T cells (memory/effector) (3 × 10^5^ cells)	In vivo	Kamanaka et al.^36^
Exacerbate colitis (*Il10* ^*−/−*^)	Colon	Disease‐prone	*Il‐10* ^*−/−*^, *Il‐10* ^*−/−*^ *Il‐22* ^*−/*−^, and *Il‐22* ^*−/−*^ mice	‐ (lack of IL‐22 shows less severe colitis)	In vivo	Gunasekera et al.^38^
Reduced microbiota	Colon	Disease‐prone	*Il‐10* ^*−/−*^, *Il‐10* ^*−/−*^ *Il‐22* ^*−/*−^, and *Il‐22* ^*−/−*^ mice	‐ (elevated levels of AMPs in *Il‐10* ^*−/−*^mice, but normalized in *Il‐10* ^*−/−*^ *Il‐22* ^*−/*−^, and *Il‐22* ^*−/−*^ mice)	In vivo	Gunasekera et al.^38^
Tumor angiogenesis promotion	Choroidal tissue	Healthy	Murine choroid explant	5/10/25 ng/mL	Ex vivo	Protopsaltis et al.^48^
Increased tumor volume	Lymphoma tumor?	Diseased	*Rag* ^*−/−*^ and C57BL/6 mice	6/12/25 mg/kg anti‐IL‐22 antibody	In vivo	Protopsaltis et al.^48^

Abbreviation: TEER, transepithelial electrical resistance.

## 4 DISCUSSION

Intestinal homeostasis depends on the intricate signaling network between gut microbiota, the host's immune system, and the intestinal epithelium integrity. It is clear that even with the knowledge there is about IL‐22 today, the result of IL‐22 signaling in intestinal health is still not completely understood. The fact that IL‐22 is produced by plethora of immune cells and has effects on several pathways in different organs indicates that this cytokine and its production must be strictly regulated.

Several unresolved issues remain in the current literature including the effect of IL‐22 on the ISC compartment, the differential effect of innate and adaptive sources of IL‐22, the presence of many regulating factors that determine the availability of IL‐22 to bind its receptor and the role of the intestinal microbiota that can induce IL‐22 and is subsequently influenced by downstream effects of IL‐22.

### 4.1 IL‐22 and epithelial proliferation

Most studies seem to agree on the fact that IL‐22 increases proliferation of intestinal epithelial cells.^8^, ^17^, ^18^, ^20^ This feature of IL‐22 certainly makes it beneficial in the mammalian intestine since the rapid renewal of the epithelium is the foundation of the symbiotic relationship between gut microbiota and the host. Without the fast regeneration of the epithelium, the intestinal microbes would likely overwhelm the host and its immune system. As IL‐22 appears to induce epithelial regeneration in the intestine, it has been suggested to use this cytokine as a therapeutic agent in dysbiotic and intestinal inflammatory disorders, such as IBD or NEC. However, the effect IL‐22 has on ISC proliferation remains unclear. Since the first observation by Lindemans et al.,^17^ others have reported that while IL‐22 increased organoid size, their survival was hampered.^18^, ^20^ In contrast, by using IL‐22 transgenic mice it was shown that IL22 increased the proliferative capacity in the TA cell compartment.^20^ In organoid culture, where ISCs might be limited upon continuous passage, this is hampering the propagation of these cultures. In vivo however, the TA compartment might be able to be replenished by the ISCs in the crypts. Zwarycz et al.^20^ suggested that IL22 might shift the balance of symmetric self‐renewal to asymmetric division where ISC division produces one ISC and one TA progenitor. More research is needed to prove this hypothesis and develop IL‐22 as a therapeutic agent to repair the intestinal barrier. Especially since He et al.,^7^ while they did observe swelling of organoids upon IL‐22 treatment, failed to see epithelial proliferation (of neither the ISC or TA compartment) in their human organoid culture. Instead, they report the induction of Paneth cells by IL‐22 in human‐derived small intestinal organoid culture. Interestingly, IL‐22 is not required for the induction of Paneth cells in mouse‐derived organoids.^50^ From these studies it is clear that translating IL‐22 effects seen in mouse models to humans might be complicated and warrants further understanding of the fundamental mechanisms of intestinal proliferation.

### 4.2 Innate versus adaptive sources of IL‐22

Another aspect that might lead to difference in IL‐22 signaling outcome is a difference in the cell types that produce IL‐22. This is well illustrated by the T‐cell transfer colitis model in mice. It has been shown that IL‐22 originating from memory/effector T cells induces pathogenicity,^36^ whereas IL‐22 produced by naïve T cells had a protective role in the T‐cell transfer colitis model.^24^ Interestingly, in the transfer of naïve T cells, IL‐22 from innate (NK/ILC) cells was suggested to next to the IL‐22 derived from CD4+ cells, also plays a protective role, since performing the transfer in *Rag1* ^−/−^ *Il22* ^−/−^ double KO mice resulted in exacerbated colitis.^24^ Next to this, temporal dynamics, the presence or absence of tissue damage and local concentrations of IL‐22 will also be important in disease promotion or inhibition. Neutrophils are among the first immune cells to arrive at the site of tissue damage and infection and are known to produce IL‐22.^5^ However, adaptive immune cells, such as T cells, arrive at the site of infection much later. It has been shown that the sequential development of innate and adaptive immunity influences IL22 signaling outcome. Innate cells (such as ILC3s) might be activated by the microbiota before an effective adaptive immune response develops.^51^ The presence of adaptive immune cells (CD4+ T cells), silenced the ILC3‐derived IL‐22‐induced pSTAT3 signaling in the epithelial cells of wildtype mice. They also showed that in Rag^−/−^ mice, in the absence of a dominant adaptive immune response, the persistent activation of ILC3s resulted in impaired lipid metabolism.

### 4.3 Presence or absence of IL‐22 regulators and cytokine context

The presence of other cytokines and regulators can affect overall immunological and pathological outcomes related to IL‐22 signaling. For example, IL‐22 together with IL‐10 may have a different effect on T‐cell activation compared with IL‐22 together with IL‐17. Next to temporal differences in IL‐22 production in the intestine, there is also spatial variability. For example, ILC3s—potent IL‐22 producers—are more abundant in the small intestine than in the large intestine.^52^ The location of IL‐22‐producing and IL‐22R‐expressing cells may therefore affect the outcome of IL‐22 signaling. Moreover, the presence of IL‐22BP in the intestine is highly important in defining the outcome of IL‐22 signaling. Additionally, the presence and the expression of IL‐22 regulators differs between intestinal sections; for example, butyrate is solely produced in the colon upon microbial fermentation. Furthermore, IL‐22BP is highly expressed in the colon compared with small intestine in healthy mice.^46^ This can significantly change the results as well as interpretations of in vivo experiments. Another important point to consider is how far‐reaching the IL‐22 signaling is. When IL‐22 production is elicited at the location of damage in one specific segment of the intestine and the cytokine remains at that site, it is likely beneficial as it induces cell proliferation and healing where needed. However, if IL‐22 moves to another site in the intestine, or other tissues, where there is no damage, it may induce needless epithelial cell proliferation, leading to possibly pathological conditions.

### 4.4 Gut microbiota and IL‐22

A host‐microbiome feedback loop also seems to exist in mice and humans in which the microbiome can influence IL‐22 production via synthesis (of precursors) of AhR ligands and IL‐22 in turn influences the microbiota composition through its actions on AMP and mucus.^53^ Alterations of AMP secretion, influences the gut microbial composition. Indeed, Zenewicz and colleagues showed that IL‐22‐deficient mice (harboring altered AMP secretion) had an altered colonic microbiota. Interestingly, this altered gut microbiota could be transmitted to cohoused wild‐type animals resulting in increased susceptibility towards colitis.

Changes in gut microbiota have an influence on the availability of metabolites.^54^, ^55^ Of special interest is the relation between tryptophan metabolism and IBD severity. Various tryptophan metabolites (such as indole derivative derived from microbial metabolism of tryptophan) can activate the AhR. Activation of AhR subsequently induces expression of IL‐22 in ILC3.^56^ A study of 535 IBD patients (both UC and CD) reported reduced serum tryptophan levels in patients with active disease.^42^ Concomitantly, increased levels of IL‐22 as well as tryptophan metabolites (quinolinic acid) were measured in serum of patients, which indicated a high activity of tryptophan degradation in patients with active IBD. Several (host and bacteria‐derived) tryptophan metabolites have been shown to influence intestinal health.^57^ Feeding tryptophan rich feeds reduced colitis in the DSS colitis mouse model and these effects were dependent on the microbiota.^57^

Taken together, it appears that when appropriately regulated, IL‐22 induces epithelial cells to secrete just the right amount of AMPs to ward off intestinal microbes at the mucosal surfaces without causing dysbiosis. However, excessive amounts of IL‐22 or too little IL‐22 may lead to overexpression or reduced expression of AMPs in the intestine that, as a consequence, trigger microbial dysbiosis possibly leading to intestinal disorders, such as UC and CD. Understanding which species of bacteria or microbial metabolites are crucial mediators of IL‐22 signaling outcome and subsequent disease might help development of novel therapeutics.

**Figure 1:**
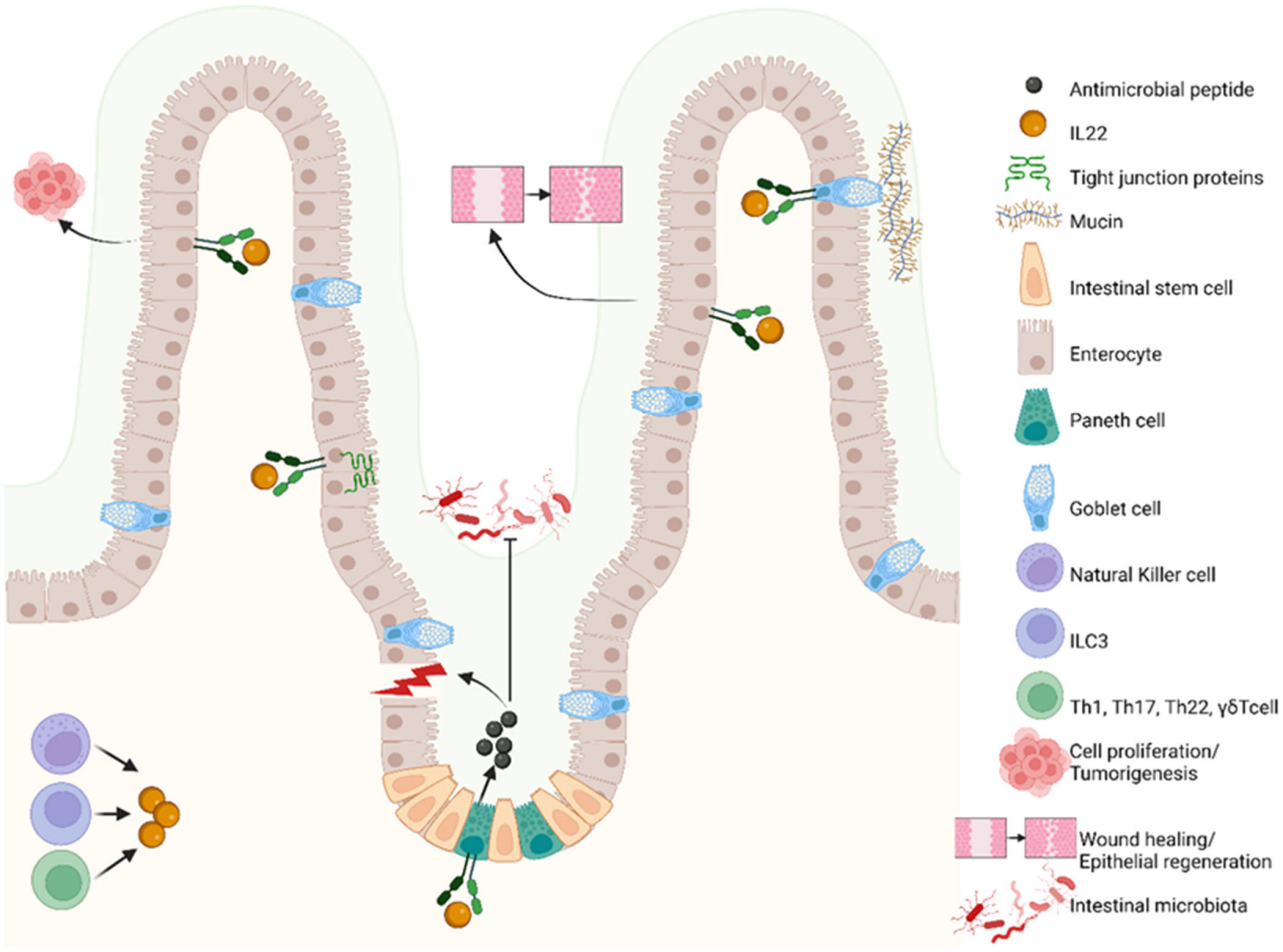
Beneficial and harmful effects of interleukin‐22 (IL22) in the intestine. IL‐22 is produced by various immune cells at the intestinal epithelium. Here, it acts on enterocytes by binding to the IL‐22 receptor complex to initiate its effects. These include inducing cell proliferation/tumorigenesis, wound healing, and induction of mucins by Goblet cells and antimicrobial peptides (AMPs) by Paneth cells. If produced in excessive amounts, these AMPs may damage the intestinal epithelium and reduce microbial diversity. IL‐22 induces tight junction proteins, modulating epithelial barrier permeability. IL‐22 also induces Paneth cells differentiation in humans. IL‐22 is tightly regulated by several factors (Box 1) (figure created with BioRender). ILC, type 3 innate lymphoid cell.

In conclusion, IL‐22 has been shown to have both positive and negative effects in the mammalian intestine, suggestive of a complex regulation of this cytokine to ensure homeostasis in the gut (summarized in Figure 1). The conditions defining the outcome of IL‐22 signaling in the intestine include the location in the intestine, the concentration of IL‐22, timing and duration, the cellular source of IL‐22, immunological context and the presence of IL‐22 regulators. IL‐22 is an appealing therapeutic agent as well as a target to help restore intestinal homeostasis, but careful consideration should be given when adopting IL‐22 or IL‐22 signaling targeted strategies as there are many conditions that define its effect in the mammalian gut.

## AUTHOR CONTRIBUTIONS

**Hedi‐Britt Klotskova**: Conceptualization, investigation, writing—original draft. **Evelien Kidess**: Writing—original draft, writing—review and editing. **Adria L. Nadal**: Visualization, writing—review and editing. **Sylvia Brugman**: Conceptualization, funding acquisition, project administration, writing—review and editing.

## Data Availability

Not applicable.
